# Anti-inflammatory nutrition with high protein attenuates cardiac and skeletal muscle alterations in a pulmonary arterial hypertension model

**DOI:** 10.1038/s41598-019-46331-4

**Published:** 2019-07-15

**Authors:** Paulien Vinke, T. Scott Bowen, Mark. V. Boekschoten, Renger F. Witkamp, Volker Adams, Klaske van Norren

**Affiliations:** 1 Nutritional Biology, Division of Human Nutrition and Health, Wageningen University, Stippeneng 4, 6708 WE Wageningen, The Netherlands; 2 University Clinic of Cardiology, Heart Center Leipzig, Strümpellstraße 39, 04289 Leipzig, Germany; 3 School of Biomedical Sciences, University of Leeds, Clarendon Way LS2 9JT, Leeds, United Kingdom; 4 Nutrition, Metabolism & Genomics Group, Division of Human Nutrition and Health, Wageningen University, Stippeneng 4, 6708 WE Wageningen, The Netherlands; 5 Laboratory of molecular and experimental cardiology, Dresden Heart Center, Fetscherstraße 76, 01307 Dresden, Germany

**Keywords:** Cardiac hypertrophy, Cardiac regeneration, Cardiovascular diseases

## Abstract

Pulmonary arterial hypertension (PAH) is characterized by remodelling of the pulmonary arteries and right ventricle (RV), which leads to functional decline of cardiac and skeletal muscle. This study investigated the effects of a multi-targeted nutritional intervention with extra protein, leucine, fish oil and oligosaccharides on cardiac and skeletal muscle in PAH. PAH was induced in female C57BL/6 mice by weekly injections of monocrotaline (MCT) for 8 weeks. Control diet (sham and MCT group) and isocaloric nutritional intervention (MCT + NI) were administered. Compared to sham, MCT mice increased heart weight by 7%, RV thickness by 13% and fibrosis by 60% (all p < 0.05) and these were attenuated in MCT + NI mice. Microarray and qRT-PCR analysis of RV confirmed effects on fibrotic pathways. Skeletal muscle fiber atrophy was induced (P < 0.05) by 22% in MCT compared to sham mice, but prevented in MCT + NI group. Our findings show that a multi-targeted nutritional intervention attenuated detrimental alterations to both cardiac and skeletal muscle in a mouse model of PAH, which provides directions for future therapeutic strategies targeting functional decline of both tissues.

## Introduction

Pulmonary hypertension (PH) is a syndrome that can result from different pathological changes in the pulmonary vasculature. PH is a progressive disease that is incurable and eventually lethal. An idiopathic or heritable form of PH, called pulmonary arterial hypertension (PAH), is the most severe form that is most commonly suffered by females. The prevalence of PAH in Europe is estimated as 15–100 per million persons, with 1- and 3-year survival rates of 87% and 67%, respectively^1,2^. The pathophysiology underlying PAH involves endothelial dysfunction of the pulmonary vasculature, metabolic abnormalities in vascular cells, hypertrophy and proliferation of smooth muscle cells, and uncontrolled growth of neointimal, medial, and adventitial layers that leads to thickening and occlusion of the small- and medium-sized pulmonary arteries^3^. Right ventricular (RV) remodeling is a major characteristic of PAH and considered to be a key mechanism driving disease progression, in which RV dysfunction is closely associated with the main symptom of exercise intolerance^1^. Other pathophysiological processes that contribute to exercise intolerance in PAH also include skeletal muscle impairments and poor nutritional status^4^.

Inflammation in particular is one key mechanism contributing to the pathological RV remodelling in PAH, which directly contributes to the development of fibrosis^5,6^. It has been consistently shown that the presence of chronic low-grade inflammation (CLGI) (*e*.*g*., elevated circulating levels of TNFα and IL-6) is linked to the development of cardiovascular disease^7^ and to changes in body composition that reduce exercise tolerance^7–9^. The use of fish oil-derived omega-3 poly-unsaturated fatty acids (PUFAs), in particular eicosapentaenoic acid (EPA) and docosahexaenoic acid (DHA) seems a promising option for the treatment of GLGI, because of their mild anti-inflammatory properties^10^. Nutritional intervention with high protein, leucine, fish oil and oligosaccharides has also been shown to have beneficial effects on skeletal muscle atrophy in a mouse model of cancer^11^. This provides evidence of a potential multi-organ treatment for PAH, where both cardiac and skeletal muscle impairments can be targeted in combination. Interestingly, the three known pathways involved in the development of PAH currently targeted by drugs^1^ include the endothelin, nitric oxide and prostacyclin pathways^12^. However while these drugs reduce pulmonary vascular resistance and improve survival, they do not always improve symptoms or address extra-cardiac maladaptations^4^. Thus while patients are living longer, their quality of life is still severely impaired due to symptoms of fatigue and exercise intolerance. A wealth of evidence in other disorders show that nutritional interventions can correct nutritional deficiencies, improve muscle function and exercise tolerance^11,13,14^. Nutritional strategies that target both cardiac and skeletal muscle remodelling in PAH (i.e., to provide a more whole-body approach) remain poorly developed, but can be of great clinical importance.

Given the adverse effects associated with PAH on multiple-organs, and the underlying role of chronic inflammation in this process, the present study aimed to test whether an anti-inflammatory nutritional intervention high in protein, fish oil and oligosaccharides could attenuate both cardiac and skeletal muscle remodelling in PAH. We induced PAH by injection of monocrotaline (MCT) for 8 weeks (a validated animal model for PAH^15,16^) in female mice to account for the current sex-dependent incidence rate of PAH in humans.

## Results

### Weight development

**Figure 1:**
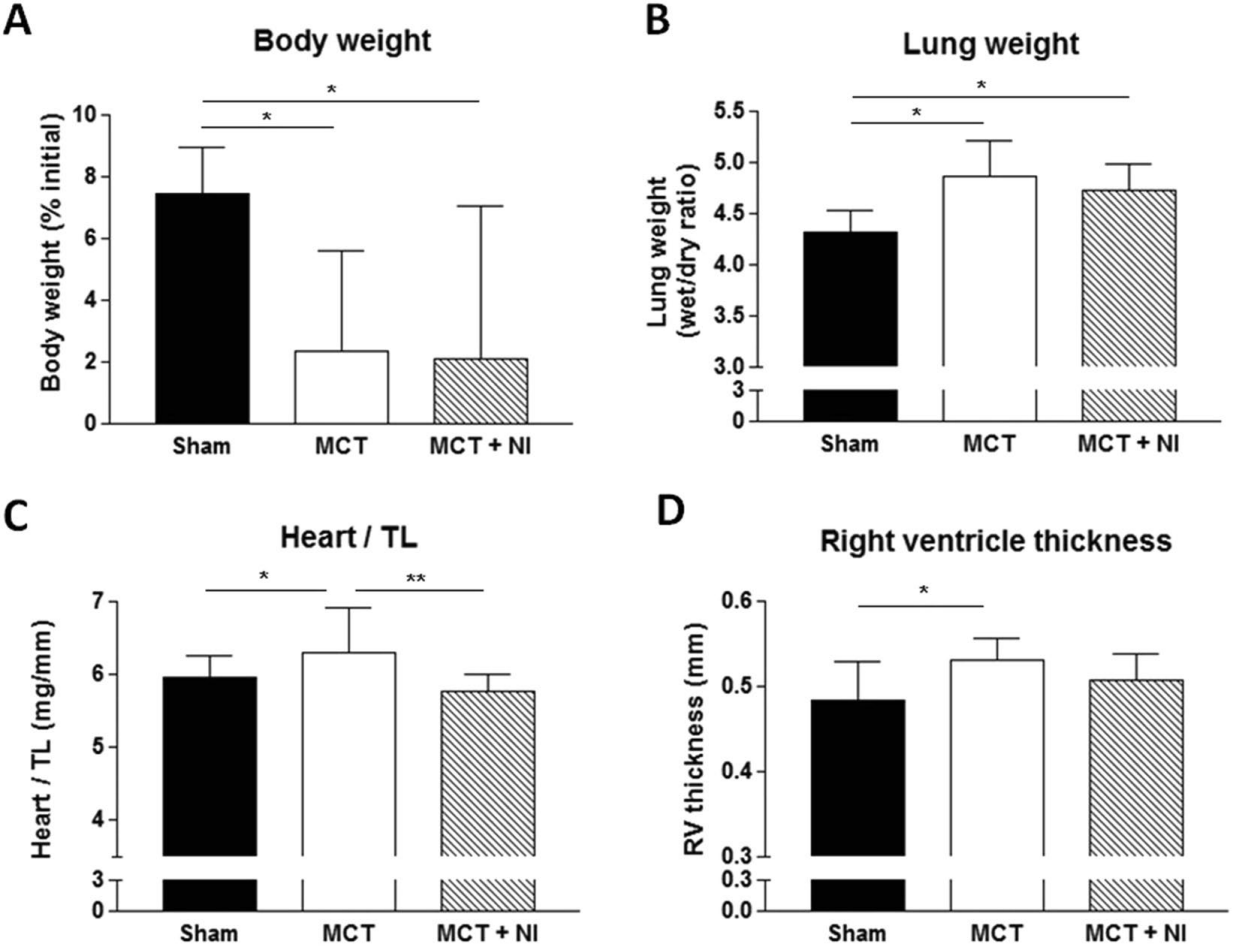
Total body weight development, lung weight and cardiac hypertrophy after 8 weeks of MCT injection. (**A**) Compared to sham mice, relative body weight was impaired (p < 0.05) in MCT and MCT + NI by 5%. (**B**) Both MCT groups showed an increase in lung weight (p < 0.05) compared to shams. (**C**) An increase in heart weight of MCT mice compared to shams was also seen, however these alterations were attenuated in MCT + NI mice. (**D**) Right ventricle thickness was increased only in MCT mice. (*p < 0.05, **p < 0.01) Scatterplots of the depicted data can be found in Supplementary Fig. S4.

Consistent with previous data^16^, MCT injections over an 8 week period resulted in a significantly reduced body weight increase over time when compared to sham animals. The increase in body weight was 7.4 ± 1.5% for sham mice, whereas MCT mice only increased weight by 2.3 ± 3.2% and MCT mice receiving nutritional intervention (MCT + NI) increased weight by 2.1 ± 4.9%: (Fig. 1A). The change in body weight was not due to a lower food intake in the MCT group compared to the sham group, as cumulative food intake of the mice was identical at the end of the intervention (see Supplementary Fig. S1).

### Development of pulmonary congestion and cardiac remodeling

Compared to sham mice, MCT mice showed an increase in pulmonary congestion (Fig. 1B), heart weight corrected for tibia length (6.30 ± 0.62 mg/mm vs 5.95 ± 0.29 mg/mm) (Fig. 1C) and right ventricular thickness (0.53 ± 0.03 mm vs 0.48 ± 0.05 mm) (Fig. 1D) (all p < 0.05). These are signs of the development of pulmonary hypertension and subsequent RV hypertrophy in the MCT group. In the MCT + NI group, heart weight was not different from sham mice and reduced compared to MCT mice (p < 0.01) (Fig. 1C). Pulmonary congestion was increased in the MCT + NI group compared to sham mice (p < 0.05) and not different from the MCT group (Fig. 1B). RV thickness in the MCT + NI group was attenuated compared to the MCT group, such that no difference was detected compared to sham mice (Fig. 1D). There were no significant changes in the thickness of the left ventricle and the septum.

### Anti-fibrotic effect of the nutritional intervention on the right ventricle

**Figure 2:**
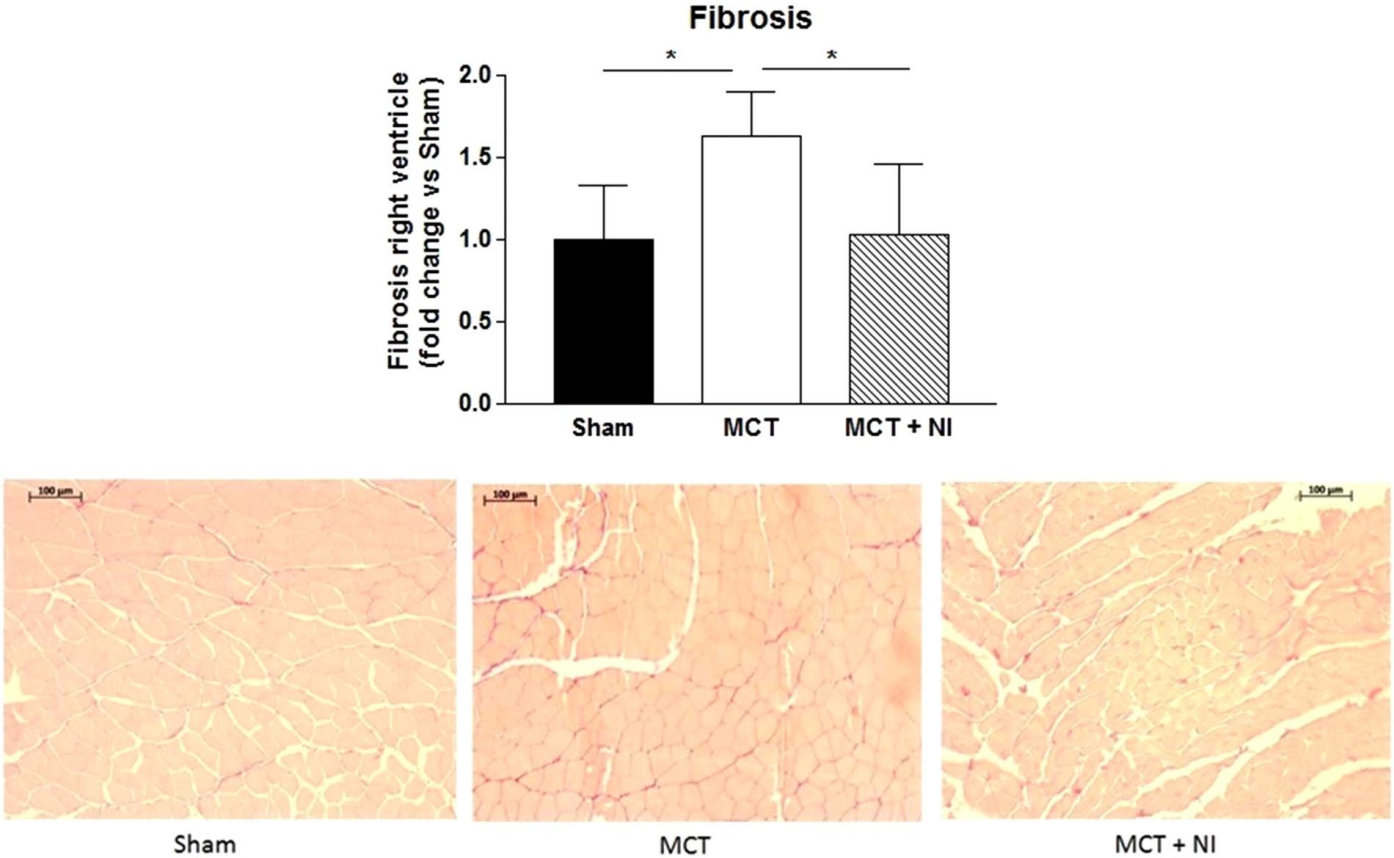
Nutritional intervention prevents increased right ventricular fibrosis after MCT injection. Right ventricular fibrosis was increased in MCT mice (p < 0.05) compared to shams and attenuated in MCT + NI mice. (*p < 0.05) A scatterplot of the depicted data can be found in Supplementary Fig. S4.

**Figure 3:**
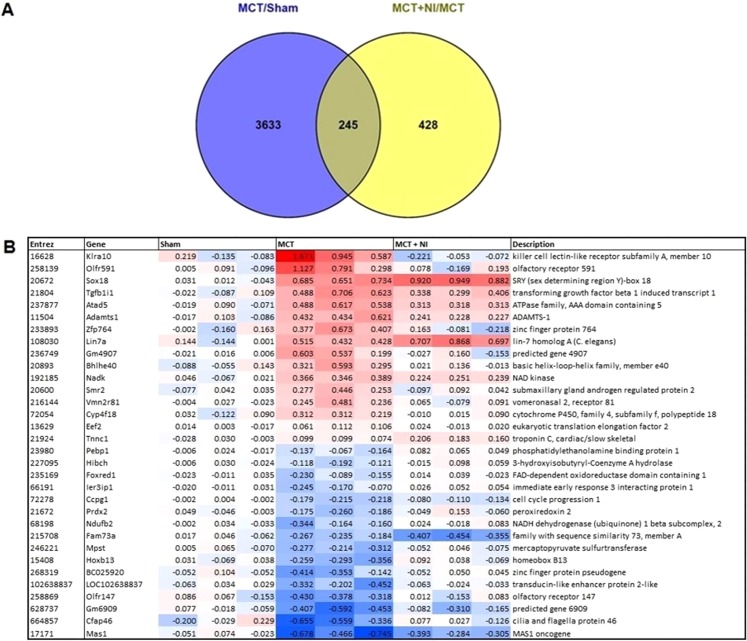
Differential expression of genes in the right ventricle after MCT injection. (**A**) A total of 245 genes were differentially expressed in MCT compared to Sham and MCT + NI compared to MCT, using a cutoff of p < 0.05. (**B**) (A) Heatmap showing 32 genes that are differentially expressed in MCT compared to sham and in MCT + NI compared to MCT, using a cutoff of p < 0.01.

Histological analysis showed a 1.6 fold change increase in fibrosis of the RV in the MCT group compared to sham (p < 0.05), that was normalized in the group receiving nutritional intervention (p < 0.05) (fold change of 1.02 versus sham) (Fig. 2A,B). Comparison of genes from the microarray analysis showed 245 genes that were differentially regulated (p < 0.05) when comparing MCT vs Sham and MCT + NI vs MCT group (Figs 3A and S2), with several of these genes involved in fibrosis. Using a cut-off of p < 0.01, 32 significantly differentially expressed genes were detected (Fig. 3B). Two of these genes are involved in fibrotic pathways (TGFb1I1 and ADAMTS-1).

Gene Set Enrichment Analysis (GSEA) of the RV of a representative subset of three mice showed overrepresentation of fibrotic pathways upon MCT injection (MCT vs. Sham) (no FDR q-value cut-off was used; the complete datasets were added as supplementary data). Upon nutritional intervention, fibrotic pathways were underrepresented in MCT + NI versus MCT (supplementary data). GSEA of the RV also showed underrepresentation of pathways involved in energy metabolism and mitochondrial function in MCT versus Sham. Upon nutritional intervention, these pathways were overrepresented (Supplementary Data).

### Validation of the microarray data on right ventricular fibrosis

**Figure 4:**
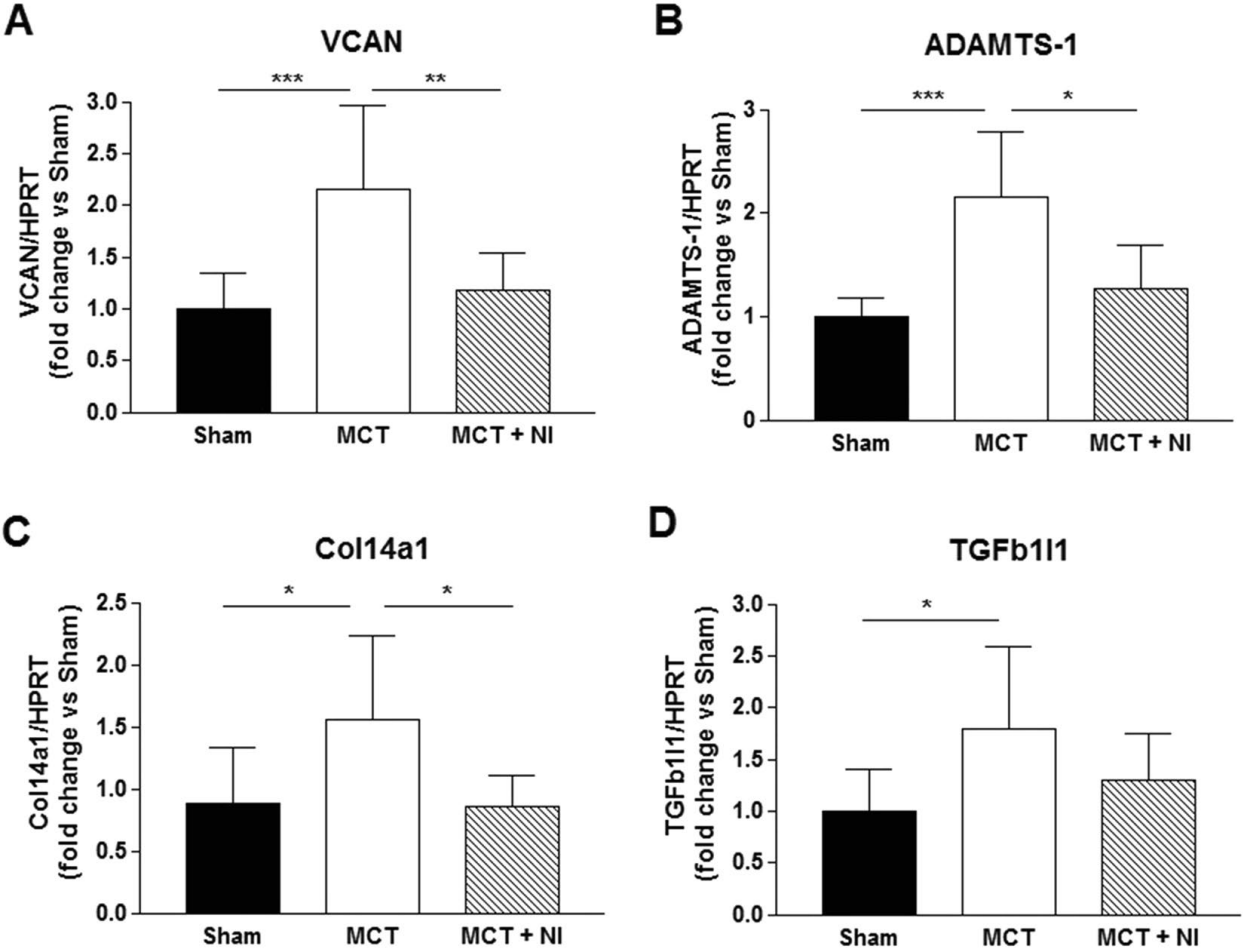
Fibrotic genes were upregulated in MCT, but not in MCT + NI goup. Compared to sham mice, mRNA levels of genes involved in fibrosis were upregulated in MCT mice, but not in MCT + NI mice (*p < 0.05, **p < 0.01, ***p < 0.001). (VCAN: versican; ADAMTS-1: a disintegrin and metalloproteinase with thrombospondin motifs 1; Col14a1: collagen type XIV alpha 1; TGFb1I1: transforming growth factor beta 1 induced transcript 1).

Next, we validated the two fibrotic genes (TGFb1I1 and ADAMTS-1) with a significance level of p < 0.01 in the microarray analysis in all our samples by qRT-PCR. We also validated two genes with a significance level of p < 0.05 (VCAN and Col14a1) by qRT-PCR. This confirmed upregulation of fibrotic genes in the right ventricle in MCT vs Sham and downregulation in MCT + NI vs MCT (Fig. 4). MCT injection induced a 2-fold increase in VCAN mRNA expression relative to sham mice (p < 0.001, Fig. 4A). In MCT-injected mice receiving nutritional intervention, this increase was 80% lower (p < 0.01, Fig. 4A). For ADAMTS-1, the effect was similar, with a 2.5-fold increase in MCT versus sham mice (p < 0.001) and only 1.25-fold increase in MCT-injected mice receiving nutritional intervention (p < 0.05, Fig. 4B). Col14a1 mRNA expression was increased 1.35-fold in MCT-injected mice relative to sham (p < 0.05), although the expression normalized in MCT mice receiving nutritional intervention (p < 0.05, Fig. 4C). mRNA expression of TGFb1I1 was increased by 65% in MCT-injected mice compared to sham (p < 0.05). The increase in MCT-injected mice receiving nutritional intervention showed no significant effect (p = 0.18, Fig. 4D).

### Effect of nutritional intervention on inflammation markers in the right ventricle

**Figure 5:**
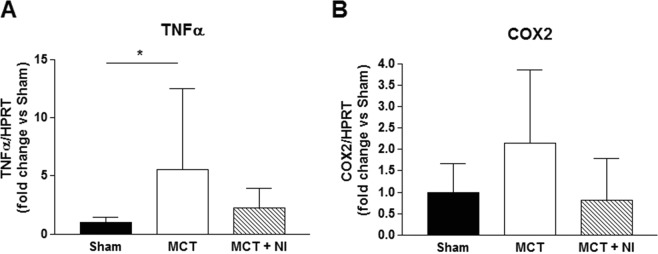
Nutritional intervention reduces an increase in TNFα mRNA levels in the right ventricle. (**A**) MCT injection induced a 5.5-fold increased level of TNFα mRNA in the right ventricle of MCT relative to sham mice. In MCT + NI mice this increase was only 2.26 fold. (**B**) COX-2 mRNA levels tended to be increased in right ventricle of MCT compared to sham mice (p = 0.068). TNFα: tumor necrosis factor alpha; COX2: cyclooxygenase-2. (*p < 0.05).

Next to higher mRNA levels of fibrotic genes, we could also show that MCT injection induced a 5.5-fold increased level of tumor necrosis factor alpha (TNFα) in the right ventricle of MCT mice relative to sham mice (p < 0.05, Fig. 5A). In MCT-injected mice receiving the nutritional intervention, this increase was only 2.26 fold (Fig. 5A). COX-2 mRNA levels also tended to be increased in right ventricle of MCT compared to sham and MCT + NI mice, although the effect was not significant (p = 0.068, Fig. 5B). These results suggest an anti-inflammatory effect of the nutritional supplement.

### Effect of nutritional intervention on the skeletal muscle

**Figure 6:**
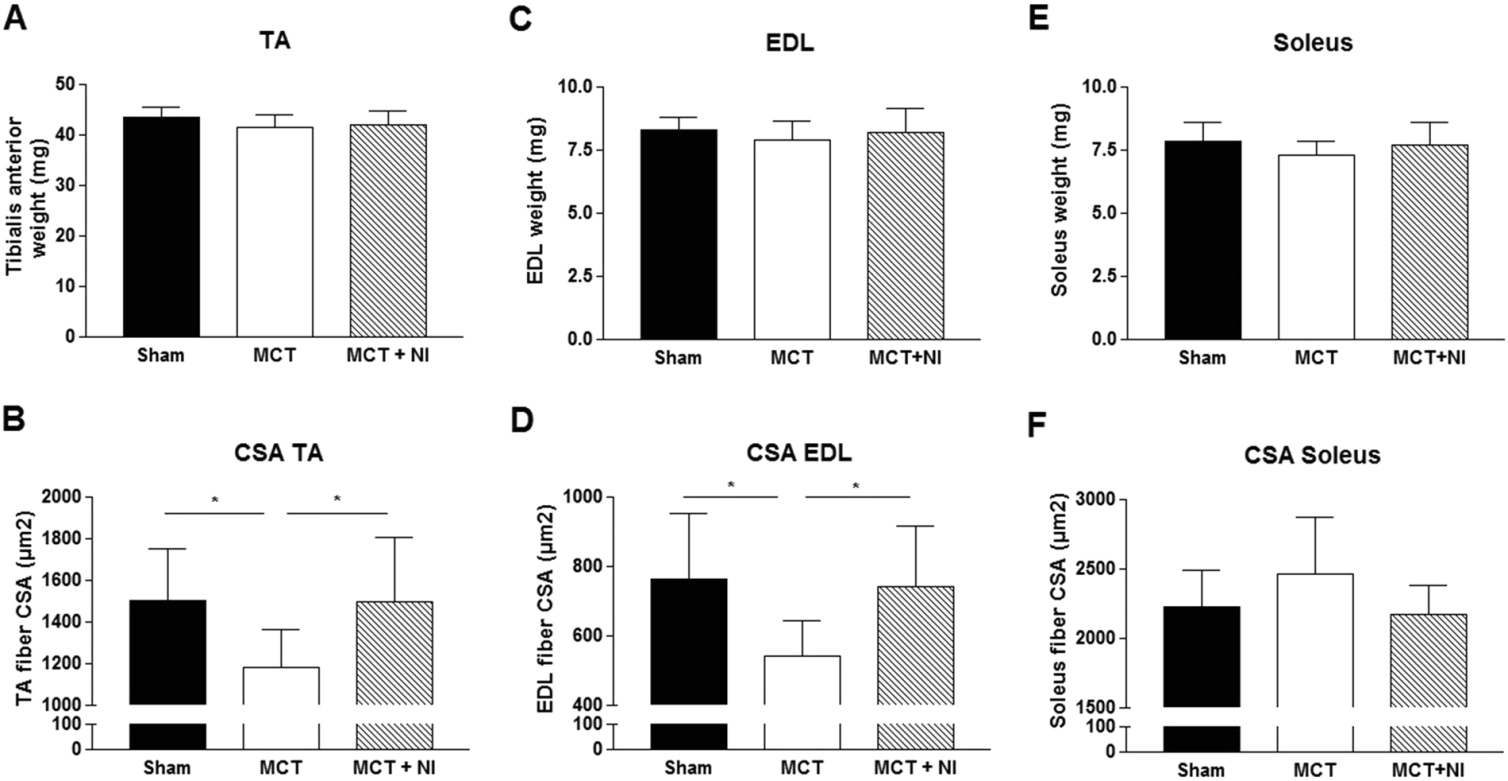
Changes in skeletal muscle weight and muscle fiber cross-sectional area. (**A**) Weight of the *tibialis anterior* (TA) muscle was unchanged, (**B**) but muscle fiber cross-sectional area (CSA) of the TA was reduced by 22% in MCT mice compared to sham, but unaltered in MCT + NI. (**B**) Weight of the EDL muscle was unchanged, but (**D**) skeletal muscle fiber CSA of the EDL was reduced by 29% in the MCT mice compared to sham and preserved in the MCT + NI group(*p < 0.05). (**E**) Weight of the soleus muscle was similar in all groups and (**F**) skeletal muscle fiber CSA was also unchanged.

**Figure 7:**
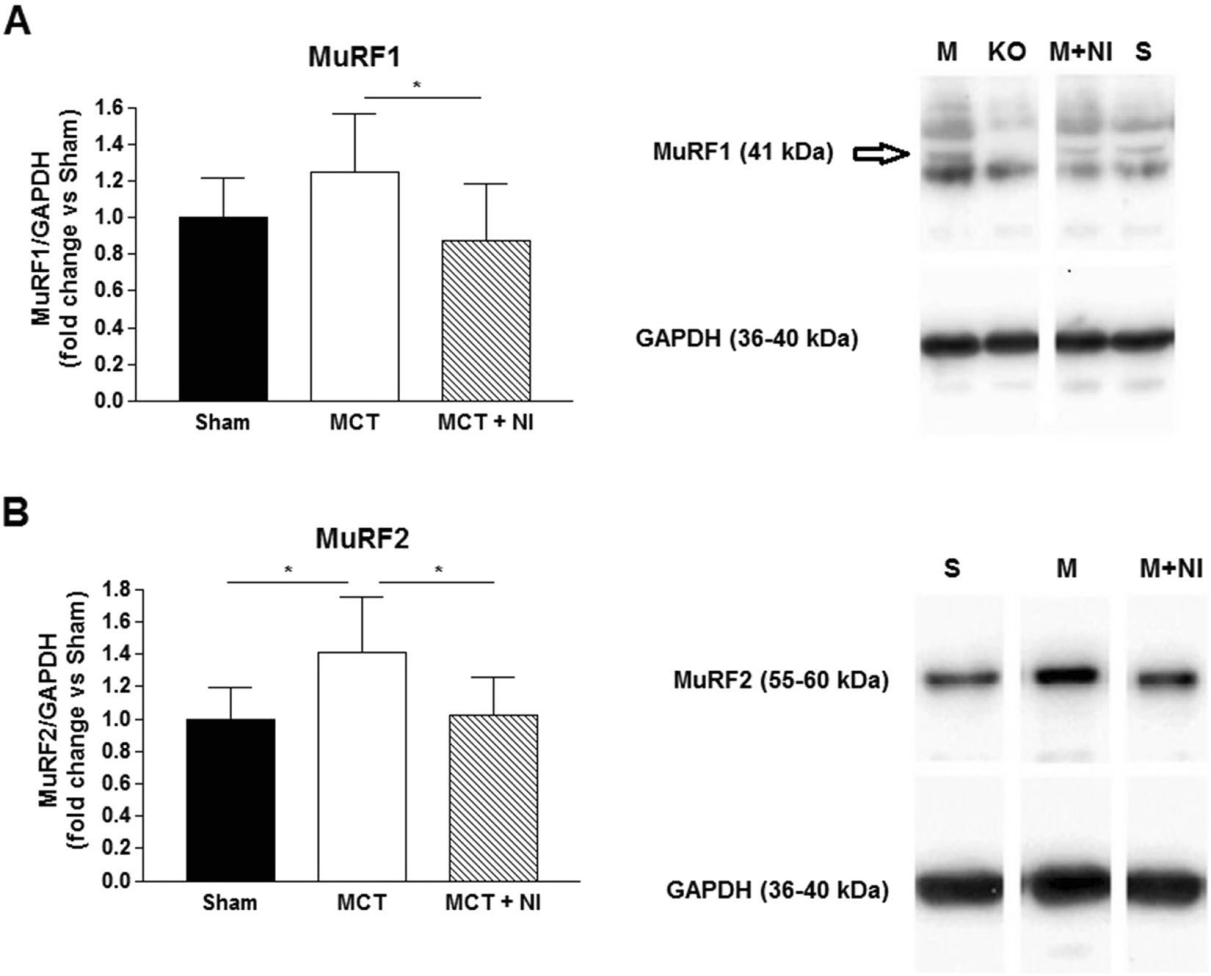
An increase in E3 ubiquitin-protein ligases MuRF1 and MuRF2 in MCT mice is prevented by nutritional intervention. (**A**) Protein levels of the key E3 ligase MuRF1 was reduced by 30% in the group receiving nutritional intervention compared to MCT mice alone. (**B**) The E3 ligase MuRF2 was increased by 41% in the MCT group compared to sham. Nutritional intervention reduced MuRF2 protein levels by 28%. (*p < 0.05). Pictures are cropped from full-length pictures of blots. Full-length blots are presented in Supplementary Fig. S3. A MuRF1 knock-out (KO) sample was included to separate the MuRF1 bands from other bands due to nonspecific binding of the antibody.

The weight of the tibialis anterior (TA) muscle was similar in all groups (Fig. 6A), however fiber cross-sectional area (CSA) of the TA muscle was reduced (P < 0.05) by 22% in MCT compared to sham mice, but preserved in the MCT + NI group (1503 vs. 1178 vs 1495 µm^2^, respectively) (Fig. 6B). Protein expression of the key E3 ligase MuRF1 was reduced by 30% in the group receiving nutritional intervention compared to MCT mice alone (p < 0.05) (Fig. 7A). The E3 ligase MuRF2 was increased by 41% (p < 0.05) in the MCT group compared to sham. Nutritional intervention reduced MuRF2 protein levels by 28% (p < 0.05) (Fig. 7B). The weight of the extensor digitorum longus (EDL) muscle was similar in all groups (Fig. 6C). However, fiber cross-sectional area was reduced by 29% (p < 0.05) in the MCT mice compared to sham, and preserved in the MCT + NI group (764 vs 542 vs 742 µm^2^, respectively) (Fig. 6D). The weight of the soleus muscle was also similar in all groups (Fig. 6E), with no effect of MCT or nutritional treatment on muscle fiber cross-sectional area (Fig. 6F).

## Discussion

The objective of this study was to assess the impact of a targeted nutritional intervention on both cardiac and skeletal muscle remodeling in a PAH model induced by MCT in mice. Indeed, adding more protein, leucine, fish oil and oligosaccharides to the feed caused an attenuation of RV hypertrophy, fibrosis and inflammation, while simultaneously preventing skeletal muscle atrophy. To our knowledge, this is the first study to show that PAH-associated pathological changes in both cardiac and skeletal muscle can be attenuated simultaneously by multiple-target nutritional intervention.

This is of great clinical importance, since current treatment options do not target both the cardiac and skeletal muscle alterations in PAH. If nutritional intervention can prevent such pathological changes and reduce the development of exercise intolerance leading to an impaired quality of life, this is an important step forward. Further studied are needed to check whether targeted nutritional intervention can improve physical status and wellbeing in PAH patients.

Eight weekly injections with MCT induced a clear PAH phenotype in mice with RV hypertrophy. Histological analysis of the RV showed an increase in fibrotic tissue in the mice receiving MCT-injections which clearly indicates cardiac remodelling typical of PAH^3,17^. The MCT mice receiving nutritional intervention did not show increased RV hypertrophy nor fibrosis. Microarray analysis of the RV and subsequent verification of the results by qRT-PCR showed that nutritional intervention prevented an increase of mRNA levels of fibrotic genes after MCT-injection in nearly all four genes. Probably the study was not powered to detect significant changes in all the genes studied. Microarray GSEA also showed downregulation of pathways involved in energy metabolism and mitochondrial function in mice receiving MCT-injections. Metabolic remodeling is another symptom of PAH. A switch from fatty acid metabolism and oxidative phosphorylation to the energetically inefficient aerobic glycolysis (also called the Warburg effect), is often seen^17–19^. Our GSEA clearly showed the downregulation of genes in energy metabolism and mitochondrial function in MCT compared to sham mice, suggesting a metabolic switch from FA oxidation to glycolysis. It also showed that this effect was reduced in the MCT group receiving the nutritional intervention.

A possible explanation for these findings is a reduction in inflammation due to the effect of added omega 3 fatty acids EPA and DHA. The mRNA levels of TNFα in the RV of the MCT mice were higher compared to sham mice. This effect was reduced upon nutritional intervention. This theory is supported by other studies using the monocrotaline model in male rats, showing reduction of PAH symptoms when anti-inflammatory treatments were provided. Anti-inflammatory drugs such as acetylsalicylic acid (aspirin)^20,21^ or 5-aminosalicylic acid^22^ have produced improvements in right ventricular systolic pressure (RVSP), RV hypertrophy and pulmonary artery remodeling. Next to this, there are also indications that certain nutritional supplements with anti-inflammatory properties including resveratrol^23–25^ and betaine^26^ reduce the effects of PAH in the MCT rat model. Supplementation with branched chain amino-acids has been described to preserve cardiac function and prolonged survival in a cancer cachexia mouse model^27^. Moreover, a diet high in leucine provided after a myocardial infarction in mice was reported to decrease fibrosis and apoptosis, and improve cardiac structure^28^.

Injection with MCT induced a reduction of muscle fiber CSA in the TA and EDL, but did not lead to alterations in the *soleus*. This might be due to the fact that the soleus has a more oxidative phenotype, reflected by a higher content of mitochondria, which might provide a certain level of protection against muscle wasting^29^. In addition, the *soleus* is more resistant to fatigue due to a relatively lower presence of glycolytic type II (fast twitch) muscle fibers, which are predominant in the EDL and TA^30^. The nutritional intervention was able to prevent the reduction in muscle fiber CSA in TA and EDL, indicating an anti-atrophic effect. This was confirmed by a reduced upregulation of the E3 ligases MuRF1 and MuRF2, which promotes muscle wasting by increasing protein degradation via ubiquitination^31^.

Faber *et al*. reported that the same nutritional combination as used in our study reduced plasma TNFα and prostaglandin E2 (PGE_2)_ in a mouse cancer cachexia model^32^. Both PGE_2_ and TNFα have been reported to induce MURF1 induction, leading to elevated proteolysis and increased protein breakdown^8,33^. When TNFα is decreased due to attenuated inflammatory tone, protein breakdown in the skeletal muscle is decreased, explaining the effects in the TA. Furthermore, skeletal muscle protein synthesis is stimulated by branched chain amino acids, especially leucine^34^, which was added to the nutritional intervention. The effect on the skeletal muscle can be direct, by a reduction of inflammation and therefore protein breakdown in the muscle itself, or indirect by a reduction in disease severity.

Chronic low-grade inflammation (CLGI) is an important part of the pathophysiology of PAH^35^. Potential triggers for CLGI in PAH include chronic hypoxia, mechanical stretch, mitochondrial dysfunction and systemic complications such as changes in intestinal functioning (barrier function, dysbiosis etc.)^9,36,37^. Chronic inflammation and expression of pro-inflammatory cytokines such as interleukin 6 (IL-6) and TNFα are known to contribute to muscle wasting which is also seen in PAH^13,38,39^. Fish oil containing (EPA) and (DHA) can influence inflammatory processes by changing the production of pro- or anti-inflammatory cytokines^40^. Other nutritional components, such as galacto- and fructo-oligosaccharides, can restore the microbial balance in case of dysbiosis^7,41^ and also have anti-inflammatory and immune-regulating effects^42^. Galacto- and fructo-oligosacharides have been shown to be able to restore T cell function during chronic inflammation in a murine tumor model when combined with omega-3 fatty acids and high quality and quantity protein^32^. Increased intake of high quality protein and the anabolic amino acid leucine can also help prevent muscle wasting^11,34^, which is why the combination of extra protein, leucine, fish oil and oligosaccharides was chosen for this study. It is unclear whether it is the whole combination or just a subset of the nutrients from the nutritional intervention that is responsible for the effects observed in this study. Further studies are warranted to reveal this aspect.

The model used for this study is an adapted version of our recently developed cachectic mouse model with monocrotaline-induced RV heart failure^16^. The adaptation concerns the control diet and the length of time the animals are in the experiment. The result is a model with similar RV remodeling but a more modest loss of body weight, which more closely resembles the clinical situation in PAH. The presence of both pulmonary congestion and RV hypertrophy, shows that this method produces effects comparable to those seen in the male rat model^43^ that is widely accepted as a model for PAH. RV thickness increased, but we found no change on the septum and thickness of the left ventricle, showing that the monocrotaline mostly affects the RV. An additional advantage of the present model is that it concerns female animals, which is more in line with the notion that PAH occurs mostly in women^1,3^.

## Conclusion

A multi-compound nutritional intervention providing higher amounts of protein, leucine, fish oil and oligosaccharides significantly attenuated cardiac and skeletal muscle changes in a female mouse model of pulmonary hypertension. The effect on the development of fibrosis in the RV was the most profound and is at least partly due to anti-inflammatory effects mediated via TNFα and COX2. These results provide directions for further study to develop novel therapeutic strategies to prevent pathophysiological alterations in pulmonary hypertension.

## Materials and Methods

### Experimental diets

The experimental diet was designed to test the effect of the combined nutritional intervention consisting of extra protein, leucine, oligosaccharides and fish oil added to the background diet AIN-93M (Research Diet Services, Wijk bij Duurstede, the Netherlands). The control diet was isocaloric to the experimental diet, and contained per kg feed 110,3 g protein (casein), 53,6 g fat (corn oil), and 653,1 g carbohydrates. The isocaloric experimental diet (further referred to as Nutritional Intervention; NI) contained per kg feed: 210 g protein (189 g intact protein of which 68% casein and 32% whey and 21 g free leucine), 57 g fat (20.2 g corn oil, 10.2 g rapeseed oil, and 22.2 g fish oil (providing 6.9 g EPA and 3.1 g DHA)), 561 g carbohydrates, 18 g galacto-oligosaccharides and 2 g fructo-oligosaccharides. Both diets were supplied as pellets.

### Animals and study design

All experiments and procedures were approved by the local Animal Research Council, University of Leipzig and the Landesbehörde Sachsen (TVV 39/17). All experiments were performed in accordance with relevant guidelines and regulations.

This study included three groups of female C57BL/6 mice (aged 8 weeks) mice: 1) saline-treated (sham; n = 9); 2) monocrotaline (MCT)-treated fed standard semi-synthetic diet AIN-93M (MCT; 600 mg/kg; n = 9); and 3) MCT-treated fed an isocaloric semi-synthetic diet with high protein, leucine, oligosaccharides and fish oil (MCT + NI; n = 10). MCT was given weekly by subcutaneous injection and sham mice received a matched volume of saline during 8 weeks. During that time period, MCT is known to induce pulmonary hypertension and RV dysfunction^15,16^. Mice were exposed to identical conditions under a 12:12 h light/dark cycle with food and water provided ad libitum. All groups received their treatment diet one week prior to the MCT injections. Body weight and food intake were recorded every week. Mice were sacrificed following deep anesthetization with i.p. administration of fentanyl (0.05 mg/kg), medetomidine (0.5 mg/kg), midazolam (5 mg/kg) and ketamine (100 mg/kg). At sacrifice, the heart, lungs, *tibialis anterior* (TA), *soleus* and *extensor digitorum longus* (EDL) were dissected, cleaned, blotted dry and weighed and *tibia* length was measured. A medial section of the heart was fixed in 4% PBS-buffered formalin. The RV was dissected from the left ventricle and immediately frozen in liquid N_2_ for RNA and protein analysis. The lungs were dried at 37 °C to measure wet/dry lung weight.

### Tissue analyses

#### RNA isolation and quantification of mRNA expression

Total RNA was isolated from RV tissue and reverse transcribed into cDNA using random hexamers and Sensiscript Reverse Transcriptase (Qiagen, Hilden, Germany). An aliquot of the cDNA was used for quantitative RT-PCR. The expression of specific genes was normalized to the expression of hypoxanthin phosphoribosyltransferase (HPRT) mRNA. For quantification of expression of specific genes, primers listed in Table 1 were used. For tumor necrosis factor alpha (TNFα) commercial primers for SYBR Green assay were used (PrimePCR, Biorad laboratories).

**Tabel 1:** Sequences of quantitative RT-PCR primers.

Symbol	Forward primer sequence (5′-3′)	Reverse primer sequence (5′-3′)
HPRT	CTCATGGACTGATTATGGACAGGAC	GCAGGTCAGCAAAGAACTTATAGCC
ADAMTS-1	AAGTGAAGCCAGCCAGTACCA	TCCCGCAAGTTTTGGAACA
VCAN	ATCAATGGGAAGCAGCTCGTT	GCATGGTAGTTGACGATTCTGT
TGFb1I1	GCCTCTGTGGCTCCTGCAATAAAC	CTTCTCGAAGAAGCTGCTGCCTC
COL14a1	CAAAAATTCTGAGCCGCTAGTT	GAGTTTCCATTTGGTCCTCTTG
COX-2	TGAGCAACTATTCCAAACCAGC	GCACGTAGTCTTCGATCACTATC

### Western blot analysis

Frozen TA muscle samples were homogenized in relax buffer (90 mmol/L HEPES, 126 mmol/L KCl, 36 mmol/L NaCl, 1 mmol/L MgCl, 50 mmol/L EGTA, 8 mmol/L ATP,10 mmol/L creatine phosphate; pH 7.4) containing a protease inhibitor mix (inhibitor mix M, Serva, Heidelberg,Germany) and sonicated. Protein concentration was determined (bicinchoninic acid assay, Pierce, Bonn,Germany), and aliquots (5 μg) were separated by SDS-polyacrylamide gel electrophoresis (10% gels). Proteins were transferred to a polyvinylidene fluoride membrane and incubated overnight at 4 °C with the following primary antibodies: MuRF1 (1/1000, Myomedix Ltd., Neckargemünd, Germany) or MuRF2 (1/1600, Myomedix Ltd., Neckargemünd, Germany). Membranes were subsequently incubated with a horseradish peroxidase-conjugated secondary antibody and specific bands visualized by enzymatic chemiluminescence (Super Signal West Pico, Thermo Fisher Scientific Inc.,Bonn, Germany) and densitometry quantified using a 1D scan software package (Vision-Capt, Vilber Lourmat, Eberhardzell, Germany). Blots were then normalized to the loading control GAPDH (1/30 000; HyTest Ltd., Turku, Finland).

### Histology

Paraffin-embedded medial cross sections (3 μm) of the heart, TA, *soleus* and EDL were mounted on glass slides. Subsequently, slides of the heart were stained with H&E and Picrosirius red to assess right ventricle wall thickness and fibrosis by imaging software (ImageJ, 1.51d, NIH and Sigma Scan Pro 5.0, Systat Software Inc). Slides of the TA and EDL were stained with H&E to assess muscle fiber cross-sectional area by imaging software (ImageJ, 1.51d, NIH). Fiber type and cross-sectional area (CSA) of the *soleus* were analyzed by immunohistochemistry; after target retrieval with Signal Stain Citrate solution (Cell Signaling), the slides were covered with proteinase K solution (Sigma, P2308), peroxidase and protein block (Dako), then incubated with anti-slow skeletal myosin heavy chain (Abcam, ab11083) in antibody dilution with background reducing factor (Dako) and anti-mouse HRP (Sigma 9044) in antibody dilution with background reducing factor (Dako). Development was performed with DAB+ coloring agent (Dako) and nuclei were colored with Mayer’s Hematoxylin Solution.

### Microarray analysis

Total RNA from the right ventricle was isolated using trizol. RNA concentrations were measured by absorbance at 260 nm (nanodrop). RNA quality was checked using the RNA 6000 nano kit assay on the Agilent 2100 bioanalyser (Agilent Technologies, Amsterdam, The Netherlands) according to the manufacturer’s protocol. For each mouse, total RNA (100 ng) was labelled using the Affymetrix WT plus reagent kit (Life Technologies, Bleiswijk, The Netherlands).

Microarray experiments were performed using Affymetrix mouse gene 1.1 ST arrays. From the experiment, 3 control samples, 3 MCT samples and 3 MCT + NI samples that were matched for disease severity were included in this experiment. Array data were analyzed using an in-house, online system^44^. Arrays passed quality control, so no samples had to be excluded from analysis. Briefly, probe sets were redefined according to Dai *et al*.^45^, using remapped computable document format version 19 based on the Entrez gene database. In total these arrays target 21.114 unique genes. Normalized expression estimates were obtained from the raw intensity values using the robust multi-array analysis (RMA) preprocessing algorithm available in the library *‘affyPLM’* using default settings^46^. Differentially expressed probe sets were identified using linear models, applying moderated t-statistics that implemented intensity-based empirical Bayes regularization of standard errors (library *‘limma’*). The moderated t-test statistic has the same interpretation as an ordinary t-test statistic, except that the standard errors have been moderated across genes, i.e. shrunk to a common value, using a Bayesian model^47,48^. Probe sets that satisfied the criterion of P < 0.01 or P < 0.05 (depending on the research question) were considered to be significantly regulated.

Changes in gene expression were related to biologically meaningful changes using gene set enrichment analysis (GSEA) of broadinstitute.org^49^. Gene sets were retrieved from the expert-curated Kyoto Encyclopedia of Genes and Genomes (KEGG), Biocarta, Reactome and WikiPathways pathway databases. Only gene sets consisting of more than 15 and fewer than 500 genes were taken into account, which resulted in the inclusion of 1,382 gene sets. For each comparison, genes were ranked on their t-value that was calculated by the empirical Bayes method. Statistical significance of GSEA results was determined using 1,000 permutations.

The array data has been submitted to the Gene Expression Omnibus (GEO)^50^, under accession number GSE125537.

### Statistical analyses

Data are presented as mean ± SD. One-way analysis of variance (ANOVA) followed by Tukey post hoc was used to compare groups (GraphPad Prism). Significance was accepted as P < 0.05.

## Supplementary information

Supplementary Information

## Data Availability

The array data generated and analysed during the current study has been submitted to the Gene Expression Omnibus (GEO)^50^, under accession number GSE125537. The GSEA datasets are available from the Supplementary Information files. Other analysed datasets are available from the corresponding author on reasonable request.
