# Supplementary material for: Anti-inflammatory nutrition with high protein attenuates cardiac and skeletal muscle alterations in a pulmonary arterial hypertension model
Source: Sci Rep. 2019 Jul 15;9:10160. doi: 10.1038/s41598-019-46331-4 (PMC6629640; doi:10.1038/s41598-019-46331-4)
Supplement: Supplementary file 1 — Supplementary Information [file 41598_2019_46331_MOESM1_ESM.pdf]

Supplementary information

Anti-inflammatory nutrition with high protein attenuates cardiac and skeletal muscle alterations in a pulmonary arterial hypertension model

Paulien Vinke [1,2] *, T. Scott Bowen [3], Mark. V. Boekschoten [4], Renger F. Witkamp [1], Volker Adams [5] §, Klaske van Norren [1] §

Affiliations

[1] Nutritional Biology, Division of Human Nutrition and Health, Wageningen University, Stippeneng 4, 6708 WE, Wageningen, the Netherlands

[2] University Clinic of Cardiology, Heart Center Leipzig, Strümpellstraße 39, 04289, Leipzig, Germany

[3] School of Biomedical Science, University of Leeds, Clarendon Way LS2 9JT, Leeds, United Kingdom

[4] Nutrition, Metabolism & Genomics Group, Division of Human Nutrition and Health, Wageningen University, Stippeneng 4, 6708 WE, Wageningen, the Netherlands

[5] Laboratory of molecular and experimental cardiology, Dresden Heart Center, Fetscherstraße 76, 01307, Dresden, Germany

[§] Contributed equally

Correspondence to: Paulien Vinke, Division of Human Nutrition and Health, Nutritional Biology Group, Wageningen University, Wageningen, the Netherlands. Stippeneng 4, 6708 WE Wageningen, The Netherlands. E-mail: paulien.vinke@wur.nl. ORCID iD: 0000-0001-6657-8065.

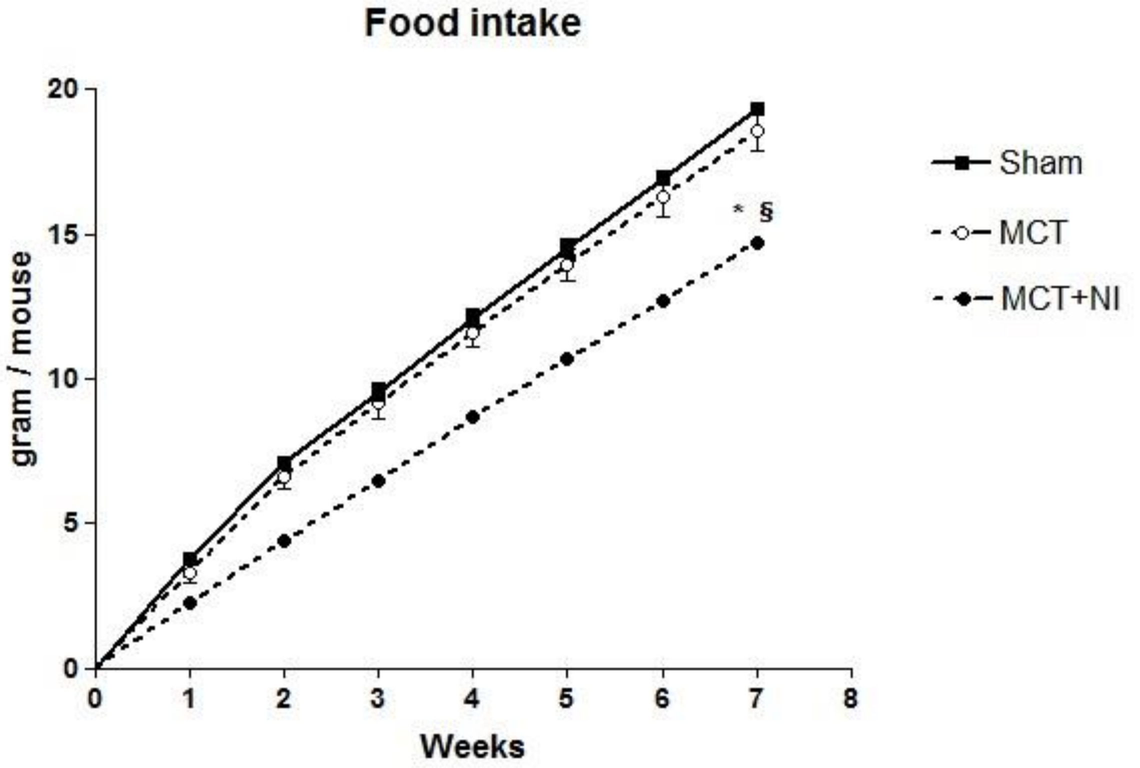

Supplementary Fig. 1: Cumulative food intake after 7 weeks was similar in sham and MCT mice, but lower in MCT+NI mice compared to MCT and sham mice. (* $p < 0.01$ versus sham; § $p < 0.05$ versus MCT)

Entrez	Gene	Control			MCT			MCT+NI			Description
15201	Hells	-0.045	0.057	-0.012	[illegible]	[illegible]	1.240	1.194	1.012	1.285	helicase, lymphoid specific
13003	Vcan	0.082	-0.253	0.171	1.233	0.893	1.197	0.870	0.365	0.835	versican
70472	Atad2	-0.103	-0.024	0.127	0.940	1.087	1.223	0.991	0.813	0.752	ATPase family, AAA domain containing 2
16628	Klra10	0.219	-0.135	-0.083	[illegible]	0.949	0.587	-0.221	-0.053	-0.072	killer cell lectin-like receptor subfamily A, member 10
192176	Flna	-0.092	-0.048	0.140	1.070	1.064	0.902	0.811	0.601	0.902	filamin, alpha
626676	Olfr471	0.091	0.530	-0.621	[illegible]	0.310	1.168	0.150	0.372	-0.463	olfactory receptor 471
239691	AU021092	0.040	0.061	-0.101	1.066	0.911	0.854	1.246	1.005	1.067	expressed sequence AU021092
15896	Icam2	-0.034	-0.053	0.086	1.007	0.893	0.836	1.039	1.015	1.122	intercellular adhesion molecule 2
66048	Aen	0.063	-0.100	0.097	0.845	0.724	0.739	0.535	0.593	0.535	apoptosis enhancing nuclease
232431	Gprc5a	0.024	0.019	-0.043	0.924	0.684	0.897	0.584	0.584	0.584	G protein-coupled receptor, family C, group 5, member A
236604	Pisd-ps1	0.033	-0.048	0.015	0.780	0.882	0.384	0.435	0.441	0.684	phosphatidylserine decarboxylase, pseudogene 1
381318	Nsl1	-0.029	-0.057	0.085	0.776	0.730	0.740	0.635	0.475	0.494	NSL1, MIND kinetochore complex component, homolog (S. cerevisiae)
258139	Olfr591	0.005	0.091	-0.096	1.127	0.791	0.298	0.078	-0.169	0.193	olfactory receptor 591
19128	Pros1	-0.076	0.017	0.057	0.676	0.794	0.715	0.837	0.885	0.913	protein S (alpha)
20672	Sox18	0.031	0.012	-0.042	0.685	0.651	0.734	0.920	0.949	0.882	SRY (sex determining region Y)-box 18
71911	Bdh1	0.312	-0.171	-0.141	0.304	0.942	0.853	1.108	1.009	1.188	3-hydroxybutyrate dehydrogenase, type 1
258000	Olfr1513	0.125	0.152	-0.277	0.560	0.812	0.511	-0.054	0.317	0.354	olfactory receptor 1513
110006	Gusb	-0.051	-0.003	0.053	0.835	0.549	0.683	0.428	0.343	0.593	glucuronidase, beta
269717	Orai2	-0.131	0.106	-0.014	0.320	0.740	0.799	0.056	-0.067	0.409	ORAI calcium release-activated calcium modulator 2
13732	Emp3	-0.091	-0.025	0.115	0.554	0.599	0.666	0.462	0.222	0.502	epithelial membrane protein 3
21804	Tgfb1i1	-0.022	-0.087	0.109	0.488	0.706	0.629	0.338	0.299	0.406	transforming growth factor beta 1 induced transcript 1
13417	Dnah8	0.167	-0.105	-0.061	0.582	0.616	0.582	0.772	0.721	0.849	dynein, axonemal, heavy chain 8
100042450	Gm18532	-0.045	-0.133	0.178	0.514	0.564	0.688	0.166	0.356	-0.435	predicted gene, 18532
258218	Olfr102	0.154	-0.140	-0.014	0.614	0.456	0.680	-0.577	0.045	0.144	olfactory receptor 102
52276	Cdca8	0.007	0.010	-0.017	0.670	0.562	0.507	0.344	0.493	0.285	cell division cycle associated 8
12818	Col14a1	0.052	-0.084	0.032	0.351	0.716	0.666	0.511	0.120	0.084	collagen, type XIV, alpha 1
29813	Bcar3	0.052	-0.122	0.070	0.770	0.508	0.415	0.335	0.335	0.335	breast cancer anti-estrogen resistance 3
56078	Car5b	0.038	-0.129	0.091	0.351	0.587	0.731	0.839	0.797	0.930	carbonic anhydrase 5b, mitochondrial
237877	Atad5	-0.019	0.090	-0.071	0.488	0.417	0.538	0.313	0.318	0.313	ATPase family, AAA domain containing 5
17330	Minpp1	-0.029	-0.153	0.183	0.629	0.579	0.423	0.431	0.160	0.185	multiple inositol polyphosphate histidine phosphatase 1
59126	Nek6	-0.023	0.007	0.016	0.587	0.528	0.502	0.345	0.345	0.457	NIMA (never in mitosis gene a)-related expressed kinase 6
29867	Cabp1	0.226	-0.039	-0.187	0.517	0.517	0.583	[illegible]	0.655	0.890	calcium binding protein 1
100126777	Esp4	0.160	-0.231	0.071	0.541	0.669	0.355	-0.388	0.160	0.093	exocrine gland secreted peptide 4
11504	Adamts1	-0.017	0.105	-0.088	0.432	0.434	0.621	0.241	0.228	0.227	a disintegrin-like and metallopeptidase (reprolysin type) with thrombospondin type 1 motif, 1
18214	Ddr2	0.129	-0.020	-0.110	0.458	0.500	0.508	0.355	0.309	0.307	discoidin domain receptor family, member 2
233893	Zfp764	-0.002	-0.160	0.163	0.377	0.673	0.407	0.163	-0.081	-0.218	zinc finger protein 764
108030	Lin7a	0.144	-0.144	0.001	0.515	0.432	0.428	0.787	0.868	0.697	lin-7 homolog A (C. elegans)
214855	Arid5a	0.117	-0.024	-0.093	0.501	0.470	0.404	0.278	0.233	0.340	AT rich interactive domain 5A (MRF1-like)
226749	Gm4907	-0.021	0.016	0.006	0.603	0.537	0.199	-0.027	0.160	-0.153	predicted gene 4907
258408	Olfr463	0.024	0.097	-0.121	0.451	0.352	0.443	0.295	-0.043	-0.035	olfactory receptor 463
20893	Bhlhe40	-0.088	-0.055	0.143	0.321	0.593	0.295	0.021	0.136	-0.013	basic helix-loop-helix family, member e40
11475	Acta2	0.034	-0.077	0.043	0.333	0.440	0.396	0.523	0.540	0.918	actin, alpha 2, smooth muscle, aorta
235442	Rab8b	-0.013	-0.007	0.020	0.414	0.404	0.336	0.242	0.234	0.343	RAB8B, member RAS oncogene family
54637	Praf2	-0.132	0.111	0.021	0.232	0.582	0.514	0.231	-0.255	0.075	PRA1 domain family 2
637896	Vmn2r78	0.119	-0.220	0.101	0.550	0.395	0.175	0.181	-0.019	-0.107	vomeronasal 2, receptor 78
319236	Trim12c	-0.050	0.039	0.011	0.528	0.417	0.165	0.884	0.409	1.006	tripartite motif-containing 12C
192185	Nadk	0.046	-0.067	0.021	0.366	0.346	0.389	0.224	0.251	0.229	NAD kinase
77011	Ticrr	0.149	-0.136	-0.012	0.434	0.368	0.291	0.186	-0.030	0.149	TOPBP1-interacting checkpoint and replication regulator
101631	Pwwp2b	-0.003	-0.038	0.041	0.277	0.402	0.403	0.212	0.242	0.252	PWWP domain containing 2B
16541	Napsa	-0.056	-0.187	0.242	0.234	0.400	0.441	-0.215	0.151	-0.039	napsin A aspartic peptidase
66713	Actr2	0.008	-0.001	-0.007	0.451	0.328	0.294	0.283	0.219	0.213	ARP2 actin-related protein 2
233246	Ano5	0.123	-0.049	-0.074	0.633	0.250	0.188	0.123	-0.120	0.123	anoctamin 5
209378	Itih5	0.066	-0.225	0.159	0.290	0.306	0.453	0.212	0.005	0.059	inter-alpha (globulin) inhibitor H5
14270	Srgap2	0.075	-0.083	0.008	0.295	0.438	0.287	0.571	0.463	0.466	SLIT-ROBO Rho GTPase activating protein 2
19697	Rela	0.062	-0.024	-0.038	0.382	0.261	0.244	0.146	0.085	0.249	v-rel reticuloendotheliosis viral oncogene homolog A (avian)
20600	Smr2	-0.077	0.042	0.035	0.277	0.446	0.253	-0.097	0.092	0.042	submaxillary gland androgen regulated protein 2
216144	Vmn2r81	-0.004	0.027	-0.023	0.245	0.481	0.236	0.065	-0.079	0.091	vomeronasal 2, receptor 81
98238	Lrrc59	-0.079	0.062	0.017	0.376	0.236	0.333	0.166	-0.019	0.221	leucine rich repeat containing 59
17691	Sik1	0.052	-0.010	-0.042	0.220	0.380	0.355	0.148	0.153	0.233	salt inducible kinase 1
226169	Pprc1	-0.075	0.111	-0.035	0.343	0.285	0.296	0.147	0.119	0.147	peroxisome proliferative activated receptor, gamma, coactivator-related 1
276905	Armc7	0.019	0.107	-0.126	0.370	0.332	0.218	0.130	0.067	0.130	armadillo repeat containing 7
70789	Kynu	0.064	0.083	-0.147	0.588	0.156	0.165	-0.051	-0.099	-0.059	kynureninase (L-kynurenine hydrolase)
381813	Prmt8	0.154	-0.103	-0.051	0.079	0.301	0.527	-0.006	0.079	-0.028	protein arginine N-methyltransferase 8
240334	Pcyox1l	-0.021	0.149	-0.128	0.332	0.258	0.316	0.139	0.130	0.117	prenylcysteine oxidase 1 like
29856	Smtn	0.009	-0.021	0.012	0.325	0.271	0.263	0.213	0.214	0.213	smoothelin
72054	Cyp4f18	0.032	-0.122	0.090	0.312	0.312	0.219	-0.010	0.015	0.090	cytochrome P450, family 4, subfamily f, polypeptide 18
226144	Erlin1	-0.122	0.091	0.031	0.400	0.155	0.273	0.091	0.091	0.091	ER lipid raft associated 1
14187	Akr1b8	-0.149	0.000	0.149	0.174	0.198	0.451	0.100	-0.021	-0.083	aldo-keto reductase family 1, member B8
435904	Igkv12-40	-0.007	0.065	-0.059	0.502	0.080	0.240	-0.016	-0.167	-0.098	immunoglobulin kappa chain variable 12-40
23821	Bace1	0.011	-0.101	0.090	0.232	0.310	0.262	0.118	0.177	0.057	beta-site APP cleaving enzyme 1
20362	39692	0.085	0.030	-0.115	0.260	0.281	0.260	0.288	0.414	0.463	septin 8
16411	Itgax	-0.025	-0.066	0.091	0.407	0.135	0.222	0.051	-0.216	-0.041	integrin alpha X
55963	Slc1a4	-0.071	0.083	-0.012	0.306	0.299	0.139	0.083	0.083	0.065	solute carrier family 1 (glutamate/neutral amino acid transporter), member 4
211225	Vmn2r15	-0.087	0.159	-0.072	0.089	0.381	0.261	-0.112	-0.196	0.021	vomeronasal 2, receptor 15
66612	Ormdl3	0.020	0.044	-0.064	0.381	0.211	0.152	-0.217	-0.010	-0.288	ORM1-like 3 (S. cerevisiae)
68344	Tmem174	0.084	0.001	-0.085	0.144	0.277	0.286	-0.078	0.148	-0.041	transmembrane protein 174
17305	Mfng	0.076	0.039	-0.115	0.249	0.396	0.055	0.544	0.582	0.436	MFNG O-fucosylpeptide 3-beta-N-acetylglucosaminyltransferase
100121	Tdrd7	-0.028	0.046	-0.018	0.268	0.239	0.158	0.570	0.424	0.322	tudor domain containing 7
243910	Nfkbid	-0.016	-0.012	0.028	0.219	0.351	0.082	0.028	0.028	0.028	nuclear factor of kappa light polypeptide gene enhancer in B cells inhibitor, delta
269941	Chsy1	-0.069	-0.014	0.083	0.145	0.262	0.210	0.083	0.060	0.090	chondroitin sulfate synthase 1
17263	Meg3	0.068	-0.130	0.062	0.183	0.252	0.182	-0.010	0.068	-0.142	maternally expressed 3
21894	Tln1	-0.010	-0.012	0.022	0.191	0.194	0.212	0.117	0.002	0.183	talin 1
74451	Pgs1	-0.024	0.095	-0.071	0.176	0.170	0.218	0.095	0.003	0.097	phosphatidylglycerophosphate synthase 1
70408	Polr3f	-0.072	0.065	0.007	0.185	0.248	0.115	0.062	0.065	0.021	polymerase (RNA) III (DNA directed) polypeptide F
216869	Arrb2	-0.001	0.037	-0.036	0.302	0.108	0.137	-0.045	-0.107	0.108	arrestin, beta 2
268564	Zbtb1	0.028	-0.016	-0.012	0.069	0.320	0.110	-0.039	0.026	-0.056	zinc finger and BTB domain containing 1
27984	Efhd2	0.030	0.002	-0.032	0.234	0.049	0.210	-0.040	-0.021	-0.076	EF hand domain containing 2
234086	Erich1	0.021	-0.074	0.053	0.134	0.157	0.159	0.232	0.337	0.200	glutamate rich 1
226278	Prlhr	0.044	-0.022	-0.022	0.153	0.164	0.164	-0.022	-0.047	-0.225	prolactin releasing hormone receptor
80383	Rbm8a	-0.064	0.056	0.008	0.213	0.066	0.165	-0.106	-0.014	0.029	RNA binding motif protein 8a
16841	Lect2	0.000	0.000	0.000	0.162	0.100	0.171	-0.092	0.000	-0.121	leukocyte cell-derived chemotaxin 2
11651	Akt1	-0.071	0.042	0.028	0.175	0.156	0.089	0.048	0.043	0.029	thymoma viral proto-oncogene 1
56324	Stam2	-0.005	-0.003	0.008	0.162	0.114	0.137	0.053	0.073	0.054	signal transducing adaptor molecule (SH3 domain and ITAM motif) 2
20397	Sgpl1	-0.001	0.040	-0.039	0.135	0.181	0.087	0.061	-0.032	0.040	sphingosine phosphate lyase 1
214469	Fam168b	-0.044	0.026	0.018	0.178	0.036	0.108	0.044	-0.110	0.021	family with sequence similarity 168, member B
101351	Eogt	-0.039	0.041	-0.002	0.041	0.176	0.106	-0.060	0.088	-0.030	EGF domain-specific O-linked N-acetylglucosamine (GlcNAc) transferase
56040	Rplp1	0.011	-0.014	0.003	0.053	0.191	0.148	0.032	0.017	0.013	ribosomal protein, large, P1
66616	Snx9	-0.025	-0.002	0.025	0.182	0.058	0.098	0.049	-0.002	0.027	sorting nexin 9
83435	Plekha5	-0.037	-0.004	0.041	0.048	0.111	0.219	0.021	0.021	-0.079	pleckstrin homology domain-containing, family A (phosphoinositide binding specific) member 5
74055	Plce1	-0.024	-0.013	0.037	0.146	0.168	0.037	0.037	0.037	-0.066	phospholipase C, epsilon 1
72102	Dusp11	-0.026	0.000	0.026	0.095	0.091	0.095	0.163	0.159	0.148	dual specificity phosphatase 11 (RNA/RNP complex 1-interacting)
13629	Eef2	0.014	0.003	-0.017	0.061	0.112	0.106	0.024	-0.013	0.020	eukaryotic translation elongation factor 2
21924	Tnnc1	-0.028	0.030	-0.003	0.099	0.099	0.074	0.206	0.183	0.160	troponin C, cardiac/slow skeletal
16783	Lamp1	-0.008	-0.051	0.059	0.084	0.096	0.090	0.155	0.245	0.221	lysosomal-associated membrane protein 1
13033	Ctsd	-0.011	-0.013	0.025	0.079	0.116	0.066	0.196	0.117	0.183	cathepsin D
26922	Mecr	0.005	-0.003	-0.003	-0.071	-0.069	-0.087	0.007	-0.003	0.086	mitochondrial trans-2-enoyl-CoA reductase
13382	Dld	0.018	-0.022	0.004	-0.107	-0.057	-0.086	0.018	0.086	-0.008	dihydrolipoamide dehydrogenase
83945	Dnaja3	-0.010	0.021	-0.011	-0.070	-0.122	-0.088	0.042	-0.018	-0.018	DnaJ (Hsp40) homolog, subfamily A, member 3
14194	Fh1	0.022	0.016	-0.038	-0.106	-0.112	-0.066	-0.052	0.045	-0.029	fumarate hydratase 1
104416	Bap1	-0.005	0.012	-0.008	-0.137	-0.075	-0.076	0.028	-0.011	-0.011	Brca1 associated protein 1
227197	Ndufs1	0.033	-0.029	-0.004	-0.040	-0.161	-0.093	-0.043	0.015	0.015	NADH dehydrogenase (ubiquinone) Fe-S protein 1
66447	Mgst3	0.009	-0.031	0.022	-0.107	-0.077	-0.114	-0.054	0.084	-0.009	microsomal glutathione S-transferase 3
72900	Ndufv2	0.014	-0.041	0.027	-0.128	-0.113	-0.063	-0.006	0.107	0.000	NADH dehydrogenase (ubiquinone) flavoprotein 2
236848	BC023829	-0.024	0.052	-0.029	-0.071	-0.080	-0.134	-0.024	-0.024	0.046	cDNA sequence BC023829
66142	Cox7b	0.042	-0.031	-0.011	-0.092	-0.096	-0.133	-0.031	0.043	-0.024	cytochrome c oxidase subunit VIIb
67851	1700021F05Rik	-0.031	0.037	-0.006	-0.142	-0.051	-0.132	-0.032	0.062	-0.024	RIKEN cDNA 1700021F05 gene
66694	Uqcrfs1	0.058	-0.029	-0.029	-0.117	-0.127	-0.084	-0.029	0.069	0.066	ubiquinol-cytochrome c reductase, Rieske iron-sulfur polypeptide 1
56282	Mrpl12	0.082	-0.037	-0.044	-0.159	-0.065	-0.108	-0.038	-0.018	0.055	mitochondrial ribosomal protein L12
14151	Fech	0.035	-0.014	-0.021	-0.204	-0.074	-0.055	-0.021	0.050	0.015	ferrochelatase
69956	Ptcd3	0.016	-0.012	-0.003	-0.092	-0.065	-0.180	0.048	0.076	-0.012	pentatricopeptide repeat domain 3
60092	Ghitm	-0.022	0.003	0.019	-0.087	-0.155	-0.096	-0.048	-0.021	-0.021	growth hormone inducible transmembrane protein
66656	Eef1d	-0.042	0.005	0.037	-0.071	-0.119	-0.165	0.014	-0.051	-0.029	eukaryotic translation elongation factor 1 delta (guanine nucleotide exchange protein)
76916	Timmdc1	-0.042	0.010	0.031	-0.138	-0.155	-0.060	-0.051	0.021	-0.034	translocase of inner mitochondrial membrane domain containing 1
73078	Pmpcb	-0.019	-0.034	0.053	-0.063	-0.146	-0.152	0.000	0.019	-0.042	peptidase (mitochondrial processing) beta
23980	Pebp1	-0.006	0.024	-0.017	-0.137	-0.067	-0.164	0.082	0.065	0.049	phosphatidylethanolamine binding protein 1
66734	Map1lc3a	0.060	-0.030	-0.030	-0.151	-0.030	-0.200	-0.030	0.061	0.076	microtubule-associated protein 1 light chain 3 alpha
22378	Wbp2	0.087	-0.088	0.000	-0.175	-0.112	-0.110	-0.334	-0.343	-0.433	WW domain binding protein 2
110821	Pcca	0.056	-0.008	-0.048	-0.159	-0.107	-0.133	-0.050	-0.015	-0.050	propionyl-Coenzyme A carboxylase, alpha polypeptide
791260	Tomt	0.000	0.000	0.000	-0.174	-0.041	-0.183	0.043	0.013	0.000	transmembrane O-methyltransferase
22318	Vamp2	0.032	-0.064	0.032	-0.122	-0.156	-0.125	-0.239	-0.234	-0.374	vesicle-associated membrane protein 2
67707	Mrpl24	-0.045	0.089	-0.045	-0.085	-0.174	-0.148	-0.045	0.033	0.023	mitochondrial ribosomal protein L24
75620	Kxd1	0.004	-0.025	0.021	-0.051	-0.201	-0.163	-0.011	-0.025	-0.072	KxDL motif containing 1
432779	Lrrc14b	-0.019	0.007	0.012	-0.094	-0.119	-0.206	-0.055	0.038	-0.018	leucine rich repeat containing 14B
13095	Cyp2c29	-0.033	-0.019	0.052	-0.071	-0.218	-0.137	-0.073	0.058	0.003	cytochrome P450, family 2, subfamily c, polypeptide 29
66865	Pmpca	-0.015	0.053	-0.039	-0.081	-0.177	-0.172	-0.037	-0.014	-0.080	peptidase (mitochondrial processing) alpha
227095	Hibch	-0.006	0.030	-0.024	-0.118	-0.182	-0.121	-0.015	0.098	0.059	3-hydroxyisobutyryl-Coenzyme A hydrolase
68263	Pdhb	-0.061	0.025	0.037	-0.190	-0.064	-0.183	-0.046	0.023	-0.058	pyruvate dehydrogenase (lipoamide) beta
78304	Naa38	0.048	-0.063	0.015	-0.161	-0.200	-0.090	-0.033	-0.013	-0.042	N(alpha)-acetyltransferase 38, NatC auxiliary subunit
235315	Rnf214	-0.011	-0.040	0.052	-0.163	-0.112	-0.176	-0.006	-0.085	-0.021	ring finger protein 214
70456	Mpc2	0.101	-0.050	-0.050	-0.136	-0.160	-0.156	-0.070	0.076	0.087	mitochondrial pyruvate carrier 2
64436	Inpp5e	0.064	-0.050	-0.014	-0.157	-0.246	-0.052	0.024	0.100	-0.050	inositol polyphosphate-5-phosphatase E
26549	Itgb1bp2	-0.026	0.028	-0.003	-0.125	-0.191	-0.148	-0.080	0.125	-0.046	integrin beta 1 binding protein 2
235169	Foxred1	-0.023	-0.011	0.035	-0.230	-0.089	-0.155	0.014	0.039	-0.023	FAD-dependent oxidoreductase domain containing 1
66191	Ier3ip1	-0.020	-0.011	0.031	-0.245	-0.170	-0.070	0.026	0.052	0.054	immediate early response 3 interacting protein 1
18036	Nfkbib	0.076	-0.018	-0.057	-0.087	-0.173	-0.240	-0.083	-0.026	-0.008	nuclear factor of kappa light polypeptide gene enhancer in B cells inhibitor, beta
75581	Yipf7	-0.048	0.087	-0.039	-0.070	-0.253	-0.196	0.062	-0.055	0.022	Yip1 domain family, member 7
18515	Pbx2	-0.041	-0.050	0.091	-0.134	-0.286	-0.116	-0.050	-0.039	0.038	pre B cell leukemia homeobox 2
230796	Wdtc1	-0.029	0.062	-0.033	-0.135	-0.215	-0.198	-0.078	-0.080	-0.078	WD and tetratricopeptide repeats 1
56451	Suclg1	0.081	-0.059	-0.022	-0.228	-0.152	-0.169	-0.076	0.004	-0.054	succinate-CoA ligase, GDP-forming, alpha subunit
100038347	Fam174b	0.017	-0.001	-0.016	-0.159	-0.159	-0.211	-0.366	-0.265	-0.233	family with sequence similarity 174, member B
68515	Myadml2	-0.002	0.038	-0.036	-0.206	-0.125	-0.220	-0.053	0.081	0.100	myeloid-associated differentiation marker-like 2
58909	Fam13a	0.040	0.003	-0.043	-0.060	-0.338	-0.190	0.016	-0.068	-0.016	family with sequence similarity 13, member A
17281	Fyco1	0.019	-0.008	-0.010	-0.140	-0.177	-0.259	-0.107	-0.106	-0.120	FYVE and coiled-coil domain containing 1
319801	9630033F20Rik	0.037	-0.069	0.032	-0.250	-0.079	-0.248	-0.043	-0.076	-0.067	RIKEN cDNA 9630033F20 gene
106821	Oard1	-0.121	0.016	0.105	-0.206	-0.257	-0.116	-0.385	-0.329	-0.491	O-acyl-ADP-ribose deacylase 1
17537	Meis3	-0.032	0.045	-0.013	-0.135	-0.207	-0.244	-0.438	-0.412	-0.239	Meis homeobox 3
258952	Olfr341	0.036	-0.042	0.007	-0.306	-0.038	-0.255	0.085	-0.016	-0.028	olfactory receptor 341
26368	Ceacam9	0.118	0.026	-0.144	-0.201	-0.179	-0.208	-0.334	-0.361	-0.517	carcinoembryonic antigen-related cell adhesion molecule 9
218341	Rfesd	-0.024	0.016	0.008	0.008	-0.310	-0.302	0.078	0.086	0.008	Rieske (Fe-S) domain containing
67811	Poldip2	0.112	-0.065	-0.047	-0.229	-0.234	-0.162	-0.115	-0.019	-0.082	polymerase (DNA-directed), delta interacting protein 2
66431	Oxld1	-0.006	-0.051	0.058	-0.122	-0.193	-0.293	-0.052	0.160	-0.051	oxidoreductase like domain containing 1
12452	Ccng2	-0.084	0.021	0.062	-0.176	-0.117	-0.318	0.009	-0.091	-0.055	cyclin G2
72278	Ccpg1	-0.002	0.004	-0.002	-0.179	-0.223	-0.218	-0.080	-0.110	-0.134	cell cycle progression 1
269514	Fbxl4	0.091	-0.102	0.011	-0.128	-0.347	-0.146	0.020	-0.050	0.086	F-box and leucine-rich repeat protein 4
107227	Macrod1	0.071	0.010	-0.081	-0.133	-0.158	-0.330	-0.084	-0.014	-0.032	MACRO domain containing 1
21672	Prdx2	0.049	-0.046	-0.003	-0.175	-0.280	-0.186	-0.049	0.153	-0.080	peroxiredoxin 2
67455	Klhl13	-0.100	0.071	0.029	-0.201	-0.293	-0.138	-0.272	-0.468	-0.416	kelch-like 13
20324	Sdpr	-0.108	0.086	0.023	-0.282	-0.133	-0.221	0.010	-0.099	-0.096	serum deprivation response
237860	Ssh2	-0.063	0.002	0.061	-0.150	-0.179	-0.307	-0.056	-0.117	0.040	slingshot homolog 2 (Drosophila)
327814	Ppfia2	-0.054	-0.044	0.098	-0.301	-0.044	-0.302	-0.044	0.005	0.012	protein tyrosine phosphatase, receptor type, f polypeptide (PTPRF), interacting protein (liprin), alpha 2
240444	Kcng2	0.031	0.080	-0.112	-0.239	-0.205	-0.184	-0.112	-0.108	-0.064	potassium voltage-gated channel, subfamily G, member 2
51869	Rif1	-0.072	0.039	0.033	-0.224	-0.214	-0.212	-0.310	-0.332	-0.318	Rap1 interacting factor 1 homolog (yeast)
13096	Cyp2c37	0.002	-0.001	-0.001	-0.207	-0.124	-0.320	0.281	-0.001	-0.001	cytochrome P450, family 2, subfamily c, polypeptide 37
268759	9930012K11Rik	-0.043	-0.043	0.085	-0.297	-0.322	-0.043	-0.043	-0.027	0.034	RIKEN cDNA 9930012K11 gene
68198	Ndufb2	-0.002	0.034	-0.033	-0.344	-0.164	-0.160	0.034	-0.016	0.083	NADH dehydrogenase (ubiquinone) 1 beta subcomplex, 2
415115	Neurl2	0.010	-0.017	0.007	-0.118	-0.235	-0.321	-0.068	-0.032	-0.049	neuralized-like 2 (Drosophila)
330657	Prss53	0.118	-0.096	-0.022	-0.176	-0.253	-0.253	-0.376	-0.436	-0.449	protease, serine 53
215708	Fam73a	0.017	0.046	-0.062	-0.267	-0.235	-0.184	-0.407	-0.454	-0.335	family with sequence similarity 73, member A
223722	Mcat	0.169	-0.084	-0.084	-0.345	-0.209	-0.136	-0.050	0.068	-0.084	malonyl CoA:ACP acyltransferase (mitochondrial)
29819	Stau2	-0.034	0.064	-0.030	-0.190	-0.237	-0.266	-0.137	-0.122	-0.129	staufen (RNA binding protein) homolog 2 (Drosophila)
258524	Olfr1168	-0.073	0.047	0.026	-0.318	-0.144	-0.238	-0.073	0.206	0.008	olfactory receptor 1168
68031	Rnf146	-0.007	0.079	-0.072	-0.183	-0.285	-0.261	-0.098	-0.092	-0.031	ring finger protein 146
72170	Chchd4	0.027	-0.048	0.021	-0.121	-0.372	-0.256	-0.087	-0.059	-0.072	coiled-coil-helix-coiled-coil-helix domain containing 4
259020	Olfr1056	-0.116	0.106	0.010	-0.332	-0.334	-0.113	0.176	-0.106	0.040	olfactory receptor 1056
20679	Sox6	-0.012	-0.059	0.071	-0.360	-0.237	-0.174	-0.372	-0.453	-0.418	SRY (sex determining region Y)-box 6
72780	Rspo3	0.076	-0.107	0.030	-0.350	-0.296	-0.139	0.056	-0.107	-0.107	R-spondin 3 homolog (Xenopus laevis)
75269	4930564D02Rik	0.197	0.035	-0.232	-0.199	-0.310	-0.277	0.160	0.346	0.321	RIKEN cDNA 4930564D02 gene
102371	Myzap	0.078	-0.030	-0.048	-0.319	-0.254	-0.229	-0.227	0.034	-0.095	myocardial zonula adherens protein
246221	Mpst	0.005	0.065	-0.070	-0.277	-0.218	-0.312	-0.052	0.046	-0.075	mercaptopyruvate sulfurtransferase
11992	Auh	0.123	-0.100	-0.023	-0.327	-0.202	-0.282	-0.129	-0.104	-0.076	AU RNA binding protein/enoyl-coenzyme A hydratase
72175	Mfsd8	-0.042	0.014	0.028	-0.324	-0.337	-0.175	-0.187	-0.109	-0.032	major facilitator superfamily domain containing 8
14202	Fhl4	-0.070	0.068	0.002	-0.374	-0.140	-0.315	-0.032	-0.059	-0.066	four and a half LIM domains 4
73451	Zfp763	-0.088	-0.049	0.137	-0.464	-0.292	-0.088	-0.088	0.109	-0.078	zinc finger protein 763
74185	Gbe1	-0.067	0.084	-0.017	-0.407	-0.281	-0.177	-0.141	-0.135	-0.111	glucan (1,4-alpha-), branching enzyme 1
74088	Them7	-0.045	0.091	-0.045	-0.186	-0.286	-0.398	-0.121	0.337	-0.045	thioesterase superfamily member 7
338374	Ifnl3	-0.161	0.101	0.061	-0.321	-0.291	-0.276	-0.547	-0.453	-0.792	interferon lambda 3
19716	Bex1	0.008	0.030	-0.038	-0.293	-0.436	-0.154	0.004	-0.091	-0.135	brain expressed gene 1
85308	Emc9	0.042	0.095	-0.137	-0.280	-0.174	-0.426	-0.143	0.008	-0.128	ER membrane protein complex subunit 9
18208	Ntn1	-0.063	-0.033	0.096	-0.420	-0.334	-0.146	-0.448	-0.454	-0.607	netrin 1
15408	Hoxb13	0.031	-0.069	0.038	-0.259	-0.293	-0.358	0.052	0.038	-0.069	homeobox B13
14525	Gcsam	-0.102	0.048	0.054	-0.070	-0.364	-0.478	-0.070	-0.046	0.207	germinal center associated, signaling and motility
268319	BC025920	-0.052	0.104	-0.052	-0.414	-0.353	-0.142	-0.052	0.050	0.045	zinc finger protein pseudogene
100101457	A830035O19Rik	0.069	-0.079	0.009	-0.406	-0.079	-0.447	-0.011	0.160	-0.079	RIKEN cDNA A830035O19 gene
27007	Klrk1	-0.064	0.093	-0.029	-0.064	-0.465	-0.412	-0.072	-0.064	-0.018	killer cell lectin-like receptor subfamily K, member 1
75712	Tmem14a	0.265	-0.001	-0.264	-0.350	-0.300	-0.300	[illegible]	-0.557	-0.761	transmembrane protein 14A
104725	Sptssa	-0.083	0.154	-0.071	-0.320	-0.194	-0.438	-0.152	-0.142	0.126	serine palmitoyltransferase, small subunit A
68270	Dnaaf1	-0.031	0.030	0.001	-0.454	-0.248	-0.259	-0.130	0.325	-0.139	dynein, axonemal assembly factor 1
102638837	LOC102638837	-0.063	0.034	0.029	-0.332	-0.202	-0.452	-0.063	-0.024	-0.033	transducin-like enhancer protein 2-like
215456	Gpat2	-0.037	0.074	-0.037	-0.441	-0.037	-0.512	0.231	-0.037	0.317	glycerol-3-phosphate acyltransferase 2, mitochondrial
14582	Gfi1b	-0.066	0.163	-0.097	-0.266	-0.559	-0.172	-0.103	0.248	-0.103	growth factor independent 1B
382985	Rrm2b	-0.101	0.148	-0.046	-0.346	-0.254	-0.400	-0.121	-0.140	-0.199	ribonucleotide reductase M2 B (TP53 inducible)
258539	Olfr830	0.146	-0.073	-0.073	-0.261	-0.246	-0.507	-0.073	-0.073	0.370	olfactory receptor 830
259041	Olfr1416	-0.145	0.166	0.000	-0.237	-0.435	-0.406	-0.170	-0.175	0.168	olfactory receptor 1416
380824	Ighv1-66	-0.127	0.006	0.121	-0.067	-0.544	-0.472	-0.063	-0.056	0.249	immunoglobulin heavy variable 1-66
258889	Olfr147	0.086	0.067	-0.153	-0.430	-0.378	-0.318	0.012	-0.153	0.083	olfactory receptor 147
14266	Aff2	-0.018	-0.110	0.128	-0.496	-0.380	-0.293	-0.770	-0.546	-0.627	AF4/FMR2 family, member 2
319885	E130114P18Rik	0.106	-0.113	0.007	-0.516	-0.298	-0.361	-0.314	-0.023	-0.126	RIKEN cDNA E130114P18 gene
215814	Ccdc28a	-0.095	-0.010	0.105	-0.287	-0.348	-0.543	-0.181	0.027	-0.201	coiled-coil domain containing 28A
436467	Trav14-1	-0.091	0.302	-0.211	-0.434	-0.338	-0.412	0.046	-0.211	0.184	T cell receptor alpha variable 14-1
66650	Nepn	-0.104	-0.029	0.133	-0.445	-0.437	-0.334	-0.583	-0.875	-0.670	nephrocan
26887	Chst6	-0.012	-0.039	0.052	-0.767	-0.376	-0.073	0.225	0.165	-0.149	carbohydrate (chondroitin 6/keratan) sulfotransferase 6
16197	Il7r	0.223	0.051	-0.275	-0.316	-0.484	-0.438	-0.085	-0.092	-0.077	interleukin 7 receptor
269016	Sh3rf2	0.332	-0.146	-0.187	-0.318	-0.352	-0.559	-0.095	0.149	-0.120	SH3 domain containing ring finger 2
54601	Foxo4	0.104	-0.026	-0.078	-0.416	-0.497	-0.359	-0.312	-0.260	-0.280	forkhead box O4
257979	Olfr1281	0.039	0.295	-0.334	-0.314	-0.419	-0.541	0.194	0.089	-0.173	olfactory receptor 1281
102632427	LOC102632427	-0.064	0.111	-0.047	-0.401	-0.460	-0.423	-0.189	-0.205	-0.261	protein FAM136A-like
74011	Slc25a27	0.041	-0.097	0.056	-0.567	-0.468	-0.256	-0.164	-0.164	-0.246	solute carrier family 25, member 27
257906	Olfr293	-0.323	0.037	0.286	-0.496	-0.323	-0.473	0.067	0.220	-0.183	olfactory receptor 293
17202	Mc4r	0.081	0.089	-0.169	-0.496	-0.276	-0.581	-0.169	0.240	-0.169	melanocortin 4 receptor
93843	Pnck	-0.007	0.004	0.004	-0.457	-0.522	-0.425	-0.443	-0.246	-0.248	pregnancy upregulated non-ubiquitously expressed CaM kinase
73736	Fcf1	0.115	-0.057	-0.057	-0.239	-0.758	-0.456	-0.291	0.014	0.106	FCF1 small subunit (SSU) processome component homolog (S. cerevisiae)
102637192	LOC102637192	-0.001	0.084	-0.084	-0.424	-0.384	-0.610	-0.373	-0.121	-0.232	keratin-associated protein 20-2-like
628737	Gm6909	0.077	-0.018	-0.059	-0.407	-0.592	-0.453	-0.082	-0.310	-0.165	predicted gene 6909
245596	Cypt2	0.170	0.088	-0.258	-0.371	-0.625	-0.342	0.113	0.044	-0.330	cysteine-rich perinuclear theca 2
664857	Cfap46	-0.200	-0.029	0.229	-0.655	-0.559	-0.336	0.077	0.027	-0.126	cilia and flagella protein 46
24050	37865	-0.350	0.193	0.157	-0.576	-0.801	-0.195	-0.891	-0.909	[illegible]	septin 3
406219	Krt83	0.391	-0.001	-0.390	-0.477	-0.630	-0.513	-0.149	-0.149	0.251	keratin 83
217666	L2hgdh	-0.098	0.123	-0.025	-0.234	-0.882	-0.669	-0.200	-0.035	-0.210	L-2-hydroxyglutarate dehydrogenase
99031	Osbpl6	-0.149	0.104	0.045	-0.682	-0.697	-0.474	-0.446	-0.265	-0.177	oxysterol binding protein-like 6
17171	Mas1	-0.051	0.074	-0.023	-0.678	-0.466	-0.745	-0.393	-0.284	-0.305	MAS1 oncogene
26442	Psma5	0.337	-0.181	-0.157	-0.895	-0.478	-0.627	-0.172	-0.261	0.250	proteasome (prosome, macropain) subunit, alpha type 5
108024	Igkv6-20	0.396	-0.394	-0.002	-0.776	-0.917	-0.415	0.027	0.489	0.230	immunoglobulin kappa variable 6-20
382395	Gm5174	-0.052	-0.192	0.243	-0.870	-0.903	-0.614	-0.489	-0.430	-0.600	predicted gene 5174
258136	Olfr517	0.006	0.194	-0.200	-1.107	-0.386	-0.845	-0.486	0.323	0.125	olfactory receptor 517
14407	Gabrg3	0.045	0.072	-0.117	-0.866	-0.877	-0.800	-0.768	-0.391	-0.543	gamma-aminobutyric acid (GABA) A receptor, subunit gamma 3

Supplementary Fig. 2: Heatmap showing 245 genes that are differentially expressed in MCT compared to sham and in MCT+NI compared to MCT, using a cutoff of $p < 0.05$.

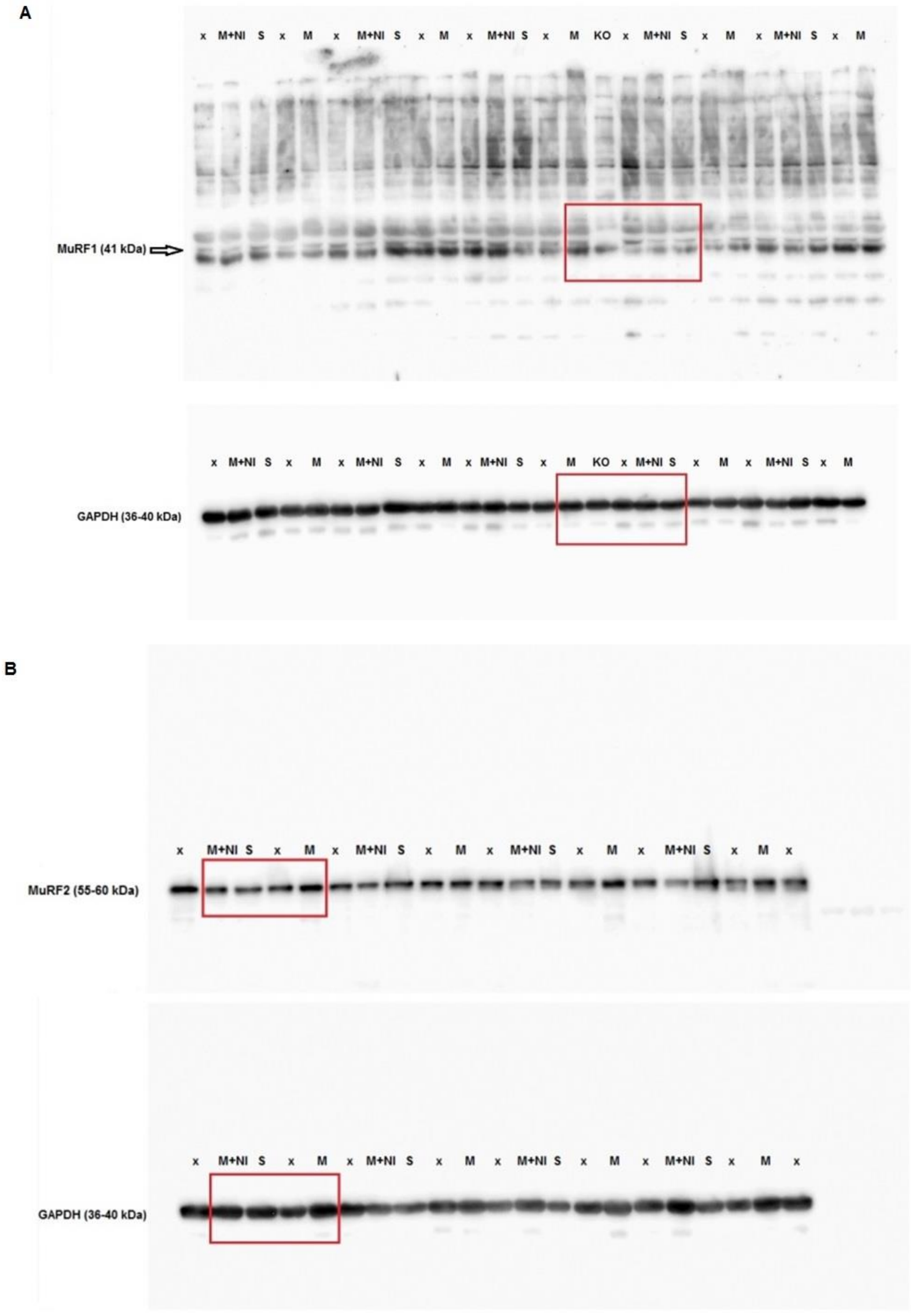
A
x M+NI S x M x M+NI S x M x M+NI S x M KO x M+NI S x M x M+NI S x M
MuRF1 (41 kDa)
x M+NI S x M x M+NI S x M x M+NI S x M KO x M+NI S x M x M+NI S x M
GAPDH (36-40 kDa)
B
x M+NI S x M x M+NI S x M x M+NI S x M x M+NI S x M x
MuRF2 (55-60 kDa)
x M+NI S x M x M+NI S x M x M+NI S x M x M+NI S x M x
GAPDH (36-40 kDa)

Supplementary Fig. 3:

A) Complete blot of MuRF1 and GAPDH on the same blot shown in figure 7A. The red boxes depict the area used in the cropped figure 7A. A MuRF1 knock-out (KO) sample was included to separate the MuRF1 bands from other bands due to nonspecific binding of the antibody. GAPDH was measured after MuRF1 incubation.

B) Complete blot of MuRF2 and GAPDH on the same blot shown in figure 7B. The red boxes depict the area used in the cropped figures in figure 7B. GAPDH was measured after MuRF2 incubation.

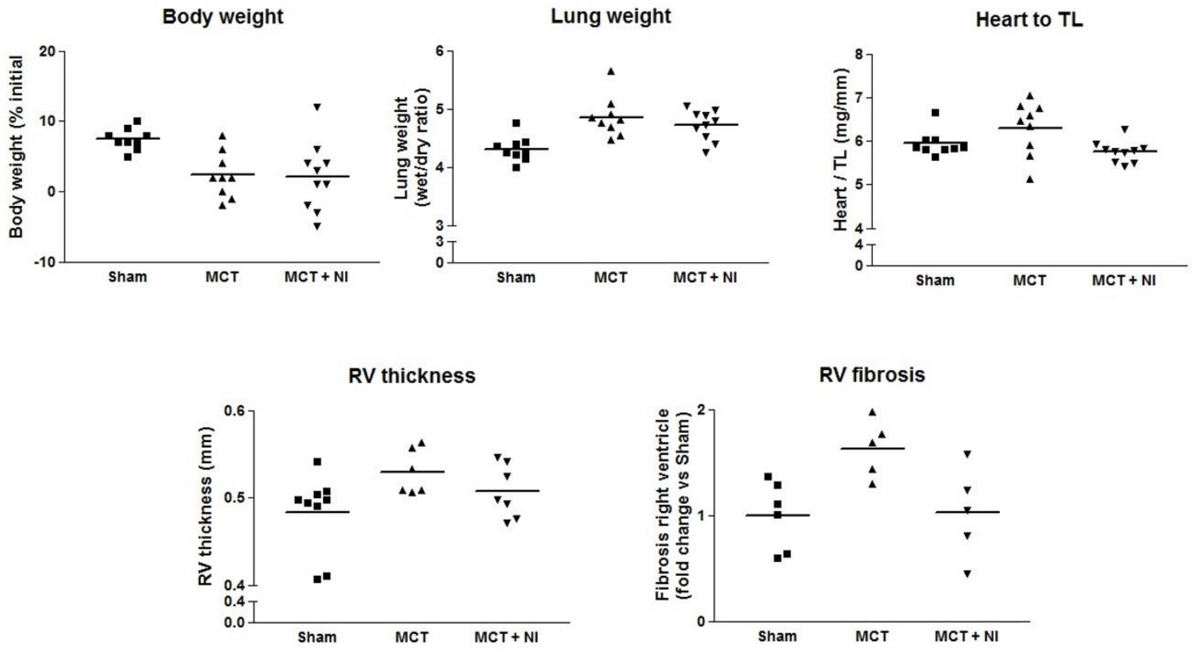

Supplementary Fig. 4: Scatterplots showing the variation of the physiological data depicted in figures 1 and 2 of the main manuscript.

Supplementary datasets 1-4 are submitted separately:

Supplementary dataset 1: GSEA Nutrition PAH RV MCT_vs_sham negative.xls

Supplementary dataset 2: GSEA Nutrition PAH RV MCT_vs_sham positive.xls

Supplementary dataset 3: GSEA Nutrition PAH RV MCT+NI_vs_MCT negative.xls

Supplementary dataset 4: GSEA Nutrition PAH RV MCT+NI_vs_MCT positive.xls
